# Effect of cachexia on bone turnover in cancer patients: a case-control study

**DOI:** 10.1186/s12885-021-08518-9

**Published:** 2021-06-28

**Authors:** Hannes Zwickl, Elisabeth Zwickl-Traxler, Alexander Haushofer, Josef Seier, Klaus Podar, Michael Weber, Klaus Hackner, Nico Jacobi, Martin Pecherstorfer, Sonia Vallet

**Affiliations:** 1 Karl Landsteiner University of Health Sciences, Dr. Karl-Dorrek-Strasse 30, Krems, 3500 Austria; 2 Department of Internal Medicine 2, University Hospital Krems, Mitterweg 10, Krems, 3500 Austria; 3 Central Laboratory, Klinikum Wels-Grieskirchen, Grieskirchner Straße 42, Wels, 4600 Austria; 4 Department of Pneumology, University Hospital Krems, Mitterweg 10, Krems, 3500 Austria; 5 IMC University of Applied Sciences Krems, Institute Krems Bioanalytics, Magnesitstraße 1, Krems, 3500 Austria

**Keywords:** Cachexia, Bone turnover, Carboxy terminal telopeptide of collagen type I (CTX), Osteocalcin (Ocn), Procollagen type I N-terminal propeptide (PINP), Hypoalbuminemia, CRP

## Abstract

**Background:**

Increased bone turnover is frequently observed in advanced cancer and predominantly related to bone metastases or therapy. Cachexia represents an important cause of morbidity and mortality in cancer patients. Key features are weight loss, muscle wasting and chronic inflammation, which induce profound metabolic changes in several organs, including the bone. However, whether cachexia contributes to abnormal bone metabolism in cancer patients is unknown. Aim of the present study was to determine the potential correlation of bone turnover markers with body composition and laboratory parameters in treatment-naïve cancer patients.

**Methods:**

In this cross-sectional study we measured the levels of carboxy terminal telopeptide of collagen (CTX), an indicator of bone resorption, as well as osteocalcin (Ocn) and procollagen type I N-terminal propeptide (PINP), indicators of bone formation, in 52 cancer patients and correlated with body composition and laboratory parameters. Univariate and multivariate logistic analysis were performed to identify determinants of negative bone remodeling balance, estimated by CTX/Ocn and CTX/PINP ratio.

**Results:**

Based on weight loss, body mass index and muscle mass, patients were divided into a cachectic (59.6%) and a control (40.4%) group. After correcting for the presence of bone metastases, our results showed a significant upregulation of CTX in cachectic patients compared to non-cachectic cancer patients (median 0.38 vs 0.27 ng/mL, *p* < 0.05), with no difference in Ocn and PINP levels (mean 14 vs. 16 ng/ml, *p* = 0.2 and median 32 vs. 26 μg/L, *p* = 0.5, respectively). In addition, the CTX/Ocn and the CTX/PINP ratio were indicative of bone resorption in 68% and 60% of cachexia patients, respectively (vs. 20% and 31% in the control group, *p* = 0.002 and *p* = 0.06). The main determinants of the unbalanced bone turnover were hypoalbuminemia for the CTX/Ocn ratio (OR 19.8, *p <* 0.01) and high CRP for the CTX/PINP ratio (OR 5.3, *p <* 0.01) in the multivariate regression analysis.

**Conclusions:**

CTX is substantially higher in cachectic patients compared to non-cachectic oncological patients and hypoalbuminemia as well as elevated CRP concentrations are independent predictors of a negative bone remodeling balance in cancer patients. These results strongly indicate that cachexia correlates with exacerbated bone turnover in cancer.

**Supplementary Information:**

The online version contains supplementary material available at 10.1186/s12885-021-08518-9.

## Background

Cachexia is a multiorgan syndrome affecting up to 80% of patients with advanced cancer. Not only is it associated with a poor quality of life, but also with high mortality rates, ranging from 20 to 80% in the first year of diagnosis [1]. Hallmarks of cancer cachexia are loss of body weight, depletion of muscle mass (sarcopenia) and systemic inflammation [2]. As a result, several tissues and organs, including adipose tissue, liver, and bone, undergo metabolic changes, which in turn contribute to cachexia in a vicious cycle.

The bone is a metabolically active organ, which undergoes a continuous remodeling mediated by a balanced activity of bone-resorbing cells, osteoclasts, and bone-forming cells, osteoblasts [3]. Changes in bone remodeling can be tracked by measuring serum levels of bone turnover markers (BTMs), including collagen breakdown products (e.g. carboxy terminal telopeptide of collagen type I (CTX)) and osteoclast-specific enzymes (e.g. acid phosphatase and cathepsin) for bone resorption; as well as by-products of collagen neosynthesis (e.g. procollagen type I N-terminal propeptide (PINP)) and osteoblast-associated proteins (e.g. bone-specific alkaline phosphatase and osteocalcin (Ocn)) for bone formation [4]. BTM serum levels strongly correlate with bone mineral density and predict risk of bone fractures [5, 6].

In cancer patients, bone damage results most frequently from skeletal metastatic disease, but is also a complication of anti-tumoral agents, hormone-targeted strategies in particular [7]. Importantly, osteopenia has also been documented in therapy-naïve patients without evidence of skeletal involvement [8, 9]. Indeed, systemic inflammation, muscle wasting, and weight loss may contribute to bone mineral loss, as observed in patients with chronic heart failure or arthritis [1, 10]. The link between cancer cachexia and increased bone turnover is supported by findings in several preclinical models, but this data has not been as yet confirmed in the clinical setting [11, 12].

To elucidate the effect of cachexia on bone turnover in cancer patients, we conducted a cross-sectional study investigating BTMs and bone regulators in treatment-naïve cancer patients with and without cachexia and their association with body composition analysis and laboratory parameters.

## Methods

### Patient population

The patient population was drawn from a recent study investigating metabolic alterations in cancer cachexia [13]. In brief, 60 adult patients with newly diagnosed cancer with or without cachexia (defined as unintentional weight loss of at least 5% during the last 6 months, or weight loss > 2% coupled with a body mass index (BMI) of < 20 kg/m^2^, or weight loss > 2% and sarcopenia [14]) were recruited at the University Hospital of Krems between February 2016 and June 2018. All patients were male and treatment-naïve to limit potential confounding factors [15]. Exclusion criteria were chronic cardiovascular diseases, insulin-dependent diabetes, or acute inflammatory state at the time of enrollment, as assessed per medical records. In addition, for the cross-sectional BTM analysis subjects with abnormal liver values due to hepatic metastases were excluded (bilirubin > 1.5 x upper limit of normal and/or GGT > 2 x upper limit of normal, *n* = 8).

Information on health and medical history was collected by interviewer questionnaires. All patients underwent clinical examination including blood pressure and anthropometric measurement (height, weight, and waist circumference), as well as routine laboratory parameters. BMI was calculated as weight in kilograms divided by height in meters squared and classified as normal (18.5–24.9 kg/m^2^), overweight (25–29.9 kg/m^2^), and obese (> 30 kg/m^2^) according to the World Health Organization recommendations

All patients provided written informed consent. The study was approved by the Ethical Committee of Lower Austria and performed according to the Declaration of Helsinki (1996).

### Body composition measurements

Analysis of body composition was performed with BodyExplorer (Juwell Medical GmbH; Rheine, Germany) following the manufacturer’s instructions. Briefly, tetrapolar single frequency (50 kHz) bioimpedance analysis (BIA) was conducted in supine patients. The following measurements were provided: I) the primary impedance parameters: resistance, reactance and phase angle, II) the algorithm-based parameters (calculated automatically by the BIA device): total body water, body cell mass (BCM), fat mass and the ratio of extracellular to intracellular water (ECW/ICW) [16]. In addition, skeletal muscle mass was estimated using the BIA equation of Janssen et al. [17]. Skeletal muscle index (SMI) was calculated by the ratio of skeletal muscle mass (kg) to squared body height (meter) and sarcopenia was defined as SMI < 14.6 kg/m^2^ [14].

### Laboratory measurements

Blood samples were collected between 8:00 and 10:00 a.m. after at least 12 h of fasting. Serum was separated immediately after phlebotomy and stored at − 80 °C until analysis. Hemoglobin, lymphocytes, creatinine, C-reactive protein (CRP), albumin, cholinesterase (CHE), transaminases, alkaline phosphatase (ALP), gamma glutamyl transferase (GGT), as well as bone-related parameters (CTX, Ocn, PINP, calcium (Ca), phosphate, thyroid stimulating hormone (TSH), 25-hydroxy vitamin D (25(OH)D) and parathyroid hormone (PTH)) were determined by well-validated laboratory routine methods. In particular, Ocn levels were assessed by a chemiluminescence immunoassay detecting intact Ocn (1–49) and large fragments (1–43). Calcium concentrations were corrected for serum albumin. The glomerular filtration rate (eGFR) was estimated using the MDRD formula.

To evaluate the balance of bone resorption and formation both the CTX/Ocn and the CTX/PINP ratio were calculated [18, 19]: values > 0.022 and > 0.011 (respective medians of the study population) indicate bone resorption.

Vitamin D status was defined as deficient for 25(OH)D concentrations < 25 ng/mL and as insufficient for 25–50 ng/mL [20].

Grouping of the variables hemoglobin, albumin, CRP and CHE was carried out using standard thresholds: hypoalbuminemia was defined as albumin < 35 g/L, high CRP as CRP > 1 mg/dL, anemia as hemoglobin < 12,5 g/dL, and low CHE as CHE < 5000 U/L [21, 22].

### Statistical analysis

Categorical variables are expressed as percentages, statistical comparisons were assessed with the Pearson’s χ^2^ test. Continuous variables were tested for normal distribution using the Shapiro-Wilk test. For comparisons of means (normally distributed variables) the Student’s t-test was used; for medians (not normally distributed variables) the Mann–Whitney U test or, in case of 3 or more variables, the Kruskal-Wallis Test were used. Correlations between CTX, Ocn, PINP and anthropometric, BIA and laboratory parameters were assessed using Spearman rank correlation coefficients.

To identify factors associated with a negative bone remodeling balance (defined as CTX/Ocn > 0.022 or CTX/PINP > 0.011), a binomial logistic regression analysis with backward stepwise approach was performed. All variables with *p*-values < 0.1 in the univariate analysis were included in the logistic regression model. Odds ratio and 95% confidence interval were used as an estimate of the risk. The Hosmer–Lemeshow test was used to assess model adequacy. Multicollinearity was assessed with variance inflation factors to confirm independence of variables included in the regression model. All statistical analyses were performed using IBM SPSS Statistics for Windows v26 (IBM Corp., Armonk, NY, USA). A *p* value of < 0.05 was considered significant.

## Results

### Patient characteristics

A total of 52 male patients with cancer at first diagnosis were included in the cross-sectional BTM study. They were further classified as cachectic (*n* = 31) or not (*n* = 21) based on the international consensus criteria for cancer cachexia [14]. The most frequent tumor types were lung cancers (73%) and gastrointestinal tumors (15%).

Table 1 summarizes the clinical characteristics of the studied population. The average age at diagnosis was 64 years. The majority of the patients (69.2%) presented with advanced stage disease (stage 3 or 4) and 15.4% had bone metastases with no statistical differences between the two groups. More than half of the patients (63.5%) presented at first diagnosis as overweight or obese, which were mainly observed in the control group (81% vs 51.6% of the patients in the cachexia group, *p* = 0.03). The 9 kg difference in body weight between the two groups was caused by a 4 kg lower BCM (28 vs 31.5, *p* = 0.01), as well as a numerically, but not statistically significant lower fat mass and total body water in cachectic patients compared to controls. Sarcopenia was observed in less than 10% of the patients, with no significant difference between cachexia and control (9.6% vs. 9.5%, *p* = 0.99). In contrast, phase angle, which is an indicator of nutritional status and muscle quality, was significantly lower in patients with cachexia (5.4 vs 6.3, *p* < 0.01) [15, 23].

**Table 1:** Patient characteristics

	All (***n*** = 52)	Cancer Cachexia (***n =*** 31)	Cancer (***n =*** 21)	***p***
**Age** ^a^	64.3 (±8.5)	64.3 (±8.3)	64.4 (±9.1)	0.9
**Stage III and IV, n (%)**	36 (69.2)	22 (71)	14 (67)	0.7
**Bone Metastases, n (%)**	8 (15.4)	3 (9.7)	5 (24)	0.2
Tumor Type^*^				0.1
**Lung, n (%)**	38 (73)	19 (61.2)	19 (90.4)	
**Gastrointestinal, n (%)**	8 (15.4)	7 (22.6)	1 (4.8)	
**Hematological, n (%)**	3 (5.8)	3 (9.7)	0 (0)	
**Other, n (%)**	3 (5.8)	2 (6.5)	1 (4.,8)	
**Anthropometric and BIA parameters**				
**BMI, kg/m**^**2** a^	26.1 (±5.1)	25.6 (±4.8)	28 (±5.1)	***0.04***
**BMI > 25 kg/m**^**2**^**, n (%)**	33 (63.5)	16 (51.6)	17 (81%)	***0.04***
**Waist circumference, cm**^a^	100.8 (±14)	98.6 (±14)	104 (±14)	0.2
**Weight, kg** ^a^	80 (±17.2)	76.3 (±17)	85.6 (±16)	0.05
**Weight loss, %** ^c^	6.5 (0–32)	10.1 (5–32)	0 (0–4)	***0.0001***
**Fat mass, kg** ^a^	20 (±9.6)	18.5 (±9.8)	22.4 (±9.2)	0.17
**Total body water, kg** ^a^	43.9 (± 6.6)	42.8 (± 6.6)	45.7 (± 6.3)	0.1
**ECW/ICW** ^a^	0.76(± 0.06)	0.76 (± 0.05)	0.75 (± 0.07)	0.4
**BCM, kg** ^a^	29.4 (±4.9)	28 (±4.4)	31.5 (± 5.1)	***0.014***
**Phase angle** ^a^	5.7 (±1.1)	5.4 (±1.1)	6.3 (±0.9)	***0.005***
**SMI, kg/m**^**2** a^	18 (±2.53)	17.9 (±2.47)	18.3 (±2.67)	0.5
**SMI < 14.6 kg/m**^**2**^**, n (%)**	5 (9.6)	3 (9.6)	2 (9.5)	0.9
**Laboratory parameters**				
**eGFR mL/min** ^b^	96.7 (79–107)	89.7 (77–109)	103 (86–107)	0.09
**CRP mg/dL** ^b^	1.1 (0.4–4.1)	2 (0.6–5.5)	0.6 (0.3–1.6)	***0.01***
**CRP > 1 mg/dl, n (%)**	27 (52)	19 (61.3)	8 (38.1)	0.1
**Albumin g/L** ^b^	36.3 (33–41)	33.9 (29–39)	39 (35–41.7)	***0.009***
**Albumin < 35 g/L, n (%)**	21 (40.3)	16 (51)	5 (23.8)	***0.04***
**CHE, U/L** ^a^	6253 (± 1984)	5629 (± 2072)	7074 (± 1512)	***0.009***
**CHE < 5000 U/L, n (%)**	11 (21.2)	10 (32.3)	1 (4.8)	***0.02***
**ALP, U/L** ^b^	81.5 (63–102)	84 (72–112)	68 (60–87)	***0.03***
**GGT, U/L** ^b^	36 (28–67)	37 (29–79)	31 (22–49)	0.09
**Hemoglobin g/dL** ^b^	14 (12–15)	13 (12–14)	14.5 (14–15.6)	***0.003***
**Hemoglobin < 12,5 g/dL, n (%)**	13 (25)	11 (35.5)	2 (9.5)	***0.03***
**Lymphocytes G/L** ^b^	1.5 (1.2–1.9)	1.4 (1–1.9)	1.6 (1.2–2.3)	0.2

***Abbreviations*:** *BIA* bioimpedance analysis, *BMI* body mass index, *ECW* extracellular water, *ICW* intracellular water, *BCM* body cell mass, *SMI* skeletal muscle index, *eGFR* estimated glomerular filtration rate, *CRP* C-reactive protein, *CHE* cholinesterase, *ALP* alkaline phosphatase, *GGT* gamma glutamyl transferase

^a^mean ± SD.

^b^median and interquartile range, 25–75th percentile.

^c^median and minimum/maximum values.

^*^gastrointestinal (GI) cancer included lower GI (2), higher GI (2), pancreato-biliary (4) tumor; hematological tumor included two myeloma and one chronic lymphocytic leukemia; other were one oropharynx, one mesothelioma and one prostate cancer.

In agreement with cachexia having been characterized by chronic inflammation [24], cachectic patients in our study presented with significantly higher CRP serum levels than in the non-cachectic control group (median 2 vs 0.6 mg/dL, *p <* 0.01). Moreover, albumin (33.9 vs 39 g/L, *p <* 0.01) and CHE (5629 vs 7079 U/L, *p* < 0.001), both indicators of hepatic function and nutritional status, were significantly reduced in cachectic patients. Interestingly, ALP serum levels were higher in patients with cachexia compared to the non-cachectic cancer control group (median 84 vs 68 U/L, *p* < 0.05), confirming recent reports of mild cholestasis in cancer cachexia [25]. Importantly, eGFR did not significantly differ between the two groups.

These observations highlight the specific clinical and laboratory features, which distinguish cachectic from non-cachectic cancer patients, including low BMI, anemia, hypoalbuminemia, and inflammation [26].

### Parameters of bone turnover and bone regulators

To elucidate the effect of cachexia on bone metabolism of cancer patients, we analyzed the levels of BTMs, CTX for bone resorption, Ocn and PINP for bone formation in particular, as well as of regulators of bone metabolism (PTH, vitamin D and TSH) based on the presence or absence of skeletal metastases (Table 2). CTX was higher in patients with cachexia compared to the non-cachectic cancer control group, but the upregulation in the cancer cachexia group gained significance after exclusion of patients with bone metastases (0.38 vs 0.31 ng/ml, *p* = 0.1 and 0.38 vs 0.27 ng/mL, *p* < 0.05). In contrast, Ocn and PINP levels did not differ between the two groups of patients. Ca levels were consistently elevated in cachectic patients, regardless of bone metastases (median 2.4 vs 2.3 mmol/L, *p* < 0.01). Interestingly, the proportion of patients with CTX/Ocn ratio above the median was significantly higher in cachectic than cancer control patients (67.7 vs 28.6%, *p <* 0.01 and 67.9 vs 18.8%, *p <* 0.01 after exclusion of bone metastases). Similarly, more patients in the cachexia group had elevated CTX/PINP ratio, although the difference did not reach statistical significance (61.3 vs 38.1%, *p* = 0.1 in the whole cohort and 60.7 vs. 31.3%, *p* = 0.06 in patients without bone metastases). Importantly, we did not observe any significant difference in BTM levels among tumor types categorized as lung or non-lung cancer or disease stages categorized as I/II, III and IV (suppl. Table 1).

**Table 2:** Markers of bone turnover and bone regulators

	All patients			Patients without bone metastases		
	Cancer Cachexia (***n =*** 31)	Cancer (***n =*** 21)	***p***	Cancer Cachexia (***n*** = 28)	Cancer (***n*** = 16)	***p***
**CTX, ng/mL**	0.38 (0.3–0.6)	0.31 (0.2–0.4)	0.08	0.38 (0.3–0.5)	0.27 (0.2–0.4)	***0.03***
**Ocn ng/ml** ^a^	14.2 (± 5.5)	16.4 (± 5.3)	0.2	14.3 (± 5.6)	16.4 (± 4.4)	0.2
**CTX/Ocn ratio > 0.022, n (%)**	21 (67.7)	6 (28.6)	***0.006***	19 (67.9)	3 (18.8)	***0.002***
**PINP, μg/L**	32 (23–39)	27.5 (22–41)	0.6	31.9 (23–38)	26.1 (21–38)	0.5
**CTX/PINP ratio > 0.011, n (%)**	19 (61.3)	8 (38.1)	0,1	17 (60.7)	5 (31.3)	0.06
**Corrected Ca, mmol/L**	2.45 (2.4–2.6)	2.35 (2.3–2.4)	***0.002***	2.43 (2.4–2.5)	2.33 (2.3–2.4)	***0.001***
**Phosphate, mg/dL**	4.2 (4–4.4)	4.3 (4.2–4.6)	0.09	4.2 (4–4.4)	4.4 (4.2–4.6)	0.1
**PTH, pg/mL**	24.1 (18–34)	27 (23–32)	0.5	24.4 (19–34)	26.6 (23–32)	0.7
**25(OH)D, ng/mL**	14.4 (8–22)	13 (8–21)	0.7	15.1 (8–22)	13 (9–21)	0.8
**TSH, μU/mL**	1.7 (1.2–3.4)	1.3 (0.7–1.9)	***0.03***	1.7 (1–3.3)	1.2 (0.6–1.7)	***0.02***

***Abbreviations*:** *CTX* carboxy terminal telopeptide of collagen type I, *Ocn* osteocalcin, *PINP* procollagen type I N-terminal propeptides, *25(OH)D* 25-hydroxy vitamin D

- All variables are expressed as median and interquartile range (25-75th percentile), except for Ocn ^a^(mean ± SD)

Except for TSH, no difference in bone regulators was observed between the two groups. Of note, in the whole cohort more than 80% of the patients had a vitamin D deficiency at first diagnosis, independently of cachexia (81% of cachectic patients vs. 90% of cancer control, *p* = 0.3). In addition, two patients (one each in the control and in the cachexia group) had inappropriately high PTH serum levels despite hypercalcemia, suggesting mild hyperparathyroidism.

In summary, the higher CTX serum levels as well as elevated CTX/Ocn and CTX/PINP ratio in cachectic patients compared to controls suggest that cachexia may exacerbate bone turnover in cancer patients.

### Association of BTM with body composition and laboratory parameters

We next evaluated the association of BTMs with body composition and laboratory parameters (Table 3). A correlation analysis revealed that BMI, body weight, waist circumference and total body water were significantly and negatively correlated to CTX (*r =* − 0.3, *p* < 0.05). In contrast, Ocn correlated only to BCM (*r =* 0.36, *p* = 0.008), as previously reported [27], and PINP to waist circumference (*r =* − 0.29, *p <* 0,05).

**Table 3:** BTM correlations with anthropometric, BIA and laboratory parameters (*n* = 52)

	CTX		Ocn		PINP	
	r	***p***	r	***p***	***r***	***p***
**Anthropometric and BIA parameters**						
**Age**	−0.04	0.7	−0.18	0.2	−0.04	0.8
**BMI**	***−0.33***	***0.01***	0.14	0.3	−0.14	0.3
**Weight**	***−0.29***	***0.03***	0.09	0.5	−0.1	0.5
**Waist circumference**	***−0.34***	***0.01***	−0.09	0.5	***−0.29***	***0.04***
**Fat mass**	−0.26	0.06	0.03	0.8	−0.1	0.4
**Total body water**	***−0.32***	***0.03***	0.05	0.7	−0.05	0.7
**ECW/ICW**	− 0.14	0.4	− 0.16	0.2	− 0.05	0.7
**BCM**	−0.19	0.2	***0.36***	***0.008***	0.06	0.6
**Phase angle**	−0.21	0.2	0.26	0.05	−0.003	0.9
**SMI**	−0.21	0.1	0.01	0.9	0.04	0.8
**Laboratory parameters**						
**eGFR**	0.07	0.6	0.2	0.1	0.07	0.6
**CRP**	***0.37***	***0.007***	−0.08	0.5	0.1	0.4
**Albumin**	***−0.45***	***0.001***	0.26	0.06	−0.12	0.4
**Cholinesterase**	***−0.39***	***0.004***	***0.37***	***0.007***	−0.1	0.5
**ALP**	0.25	0.07	0.04	0.7	0.25	0.07
**GGT**	***0.31***	***0.02***	−0.15	0.26	0.01	0.9
**Hemoglobin**	−0.27	0.05	0.17	0.2	−0.21	0.1
**Lymphocytes**	−0.17	0.2	0.06	0.6	−0.19	0.2
**Bone metabolism parameters**						
**PTH**	−0.11	0.4	0.19	0.2	−0.06	0.7
**25(OH)D**	−0.16	0.2	0.13	0.3	0.04	0.7
**TSH**	0.19	0.2	−0.06	0.6	0.07	0.6

***Abbreviations*:** *BTM* bone turnover markers, *BIA* bioimpedance analysis, *CTX* carboxy terminal telopeptide of collagen type I, *Ocn* osteocalcin, *PINP* procollagen type I N-terminal propeptides, *BMI* body mass index, *ECW* extracellular water, *ICW* intracellular water, *BCM* body cell mass, *SMI* skeletal muscle index, *eGFR* estimated glomerular filtration rate, *CRP* C-reactive protein, *ALP* alkaline phosphatase, *GGT* gamma glutamyl transferase, *25(OH)D* 25-hydroxy vitamin D

Among the laboratory parameters CTX strongly correlated with markers of inflammation, CRP (*r =* 0.39, *p* < 0.01) and albumin (*r =* − 0.45, *p <* 0.01) in particular. In addition, both CTX and Ocn correlated with CHE (CTX *r =* − 0.39 and Ocn *r =* 0.37, *p <* 0.01). Regarding bone metabolism parameters, no significant correlation with BTMs was observed. After exclusion of patients with bone metastases, significant correlations persisted between CTX and waist circumference, CRP, albumin, and CHE; Ocn correlated only with BCM; PINP correlated with waist circumference and hemoglobin levels (suppl. Table 2).

Taken together, this data indicates an association between CTX and the clinical and laboratory features characterizing cancer cachexia, such as BMI, albumin, and CRP.

### Determinants of negative bone remodeling balance

The uncoupling of bone formation and resorption results in a negative bone remodeling balance, which is considered a risk factor for osteoporosis and bone fractures [19]. By using the median of our study population as a cutoff (0.022 for CTX/Ocn and 0.011 for CTX/PINP), BTM ratio > median indicated an unbalanced bone turnover with increased bone resorption [18, 19]. Among the patients with an elevated CTX/Ocn ratio 78% were cachectic and 22% non-cachectic (*p* = 0.01). Similarly, 70.4% of the patients with increased CTX/PINP ratio were cachectic (vs. 29.6% of control, p 0.1). Importantly, after excluding bone metastatic disease the proportion of cachectic and cancer control patients among those with an elevated BTM ratio shifted to 86% vs. 14% for the CTX/Ocn ratio (*p* = 0.004) and 77.3% vs. 22.7% for the CTX/PINP ratio (*p* = 0.06).

To identify determinants of negative bone remodeling balance in cancer patients, we performed a logistic regression analysis, which revealed hypoalbuminemia for the CTX/Ocn ratio (OR of 19.8, 95% CI 3.5–111, *p* < 0.01) and high CRP for the CTX/PINP ratio (OR of 5.29, 95% CI 1.6–17.5, *p <* 0.01) as main indicators of unbalanced bone turnover in the whole patient population. After exclusion of patients with bone metastatic disease, both hypoalbuminemia and high CRP were associated with a negative bone turnover (CTX/Ocn: OR 10.5 for hypoalbuminemia and OR 7.6 for high CRP; CTX/PINP: OR 7.7 for high CRP; *p* ≤ 0.01) (Table 4).

**Table 4:** Variables associated with a negative bone turnover balance in cancer patients (*n =* 52)

	All patients (***n =*** 52)			Patients without bone metastases (***n*** = 44)		
	OR	CI	***p***	OR	CI	***p***
**CTX/Ocn ratio > 0.022**^a^						
Hypoalbuminemia	19.8	3.5–111.5	***0.001***	10.5	1.6–65.6	***0.01***
High CRP	4.56	1–20	0.05	7.6	1.5–38.4	***0.01***
Adjusted R squared	0.54			0.52		
**CTX/PINP ratio > 0.011**^b^						
High CRP	5.29	1.6–17.5	***0.006***	7.7	1.9–30.5	***0.003***
Adjusted R squared	0.2			0.3		

***Abbreviations*:** *OR* odds ratio, *CI* confidence interval, *CRP* C-reactive protein, *CTX* carboxy terminal telopeptide of collagen type I, *Ocn* osteocalcin, *PINP* procollagen type I N-terminal propeptides

- The backward stepwise regression models included:

^a^BMI, body weight loss (weight loss > 0%), hypoalbuminemia (albumin < 35 g/L), high CRP (CRP > 1 mg/dL), anemia (hemoglobin < 12,5 g/dL), phase angle, eGFR adjusted for age.

^b^Body weight loss (weight loss > 0%), hypoalbuminemia (albumin < 35 g/L), high CRP (CRP > 1 mg/dL), adjusted for age.

In summary, this data suggests that among the BIA, anthropometric and laboratory parameters, only hypoalbuminemia and high CRP were predictors of the negative bone remodeling balance of cancer patients.

## Discussion

The balance of bone homeostasis can be severely undermined in cancer patients [7]. Several treatment strategies, including chemotherapy and antihormonal agents, lead to bone loss and significantly increase the risk of fracture [28]. Skeletal metastatic disease is also a common cause of deregulated bone metabolism due to pathological cross-talk between tumor cells and the bone milieu [29]. Interestingly, osteopenia and osteoporosis have also been observed in therapy-naïve patients without evidence of skeletal involvement [8, 9], thus suggesting that additional factors other than bone metastases and treatments may affect bone turnover in cancer patients. At first diagnosis as many as 42% of oncological patients present signs of cachexia [30], a multiorgan syndrome characterized by weight loss, wasting of muscle mass and systemic inflammation; all of which are frequently associated with bone loss [31, 32]. However, the effect of cachexia on bone health in cancer patients has not been fully elucidated. In this study, we investigated bone metabolism in treatment-naïve cancer patients and showed for the first time an upregulation of the bone resorption marker, CTX, in the presence of cachexia. In addition, we identified hypoalbuminemia and high CRP as predictors of the negative bone turnover balance of cancer patients.

Skeletal metabolism is finely tuned by a complex network of local and systemic factors, including gonadal hormones, vitamin D and PTH, and derangements of these pathways may alter the delicate balance of bone remodeling. Vitamin D deficiency is a frequent finding in cancer patients and may contribute to bone loss due to secondary hyperparathyroidism [33–35]. PTH and tumor-derived PTHrP enhance bone loss by uncoupling bone resorption and formation [36]. Animal studies also suggest that PTHrp may mediate cachexia via browning of adipose tissue and subsequent increase in energy expenditure [37], but the clinical relevance of this pathway has yet to be confirmed. In our study, PTH negatively correlated with Ca levels (*r =* − 0.39, *p* < 0.01), but not with BTMs, thus excluding hyperparathyroidism as a cause of accelerated bone turnover in this patient cohort.

The presence of cachexia has been associated with osteoporosis in patients with chronic obstructive pulmonary disease, rheumatoid arthritis, and heart failure [38, 39]. Indeed, weight loss, muscle wasting, and chronic inflammation alter the balance of bone turnover by increasing bone resorption and inhibiting formation [31, 32, 40]. In our study, BTM ratio was indicative of bone resorption in more than 60% of the patients with cachexia and the main determinants of the negative bone turnover balance were hypoalbuminemia and high CRP. Importantly, hypoalbuminemia and elevated CRP are not only associated with poor outcomes in several cancer types [21, 41], but also to osteoporosis and fracture risk [42, 43].

The present study has several limitations, including the small number of included patients with different tumor types and disease stages, the absence of a control group with healthy subjects, and the lack of histomorphometric and bone density assessment. Since a negative balance of bone remodeling may lead to low bone mass, a longitudinal design with morphometric correlates and bone imaging studies is warranted to evaluate if increased bone resorption results from hyperactivation of osteoclasts and translates into osteopenia and osteoporosis. Moreover, it is important to point out that the reliability of some BIA measurements is questioned in case of obesity, large tumor mass and fluid retention. In contrast to multi-frequency instruments, single-frequency devices overestimate total body water in case of edema [44, 45]. In addition, assessment of muscle mass by BIA is inaccurate in obese patients. In our study less than 10% of the patients was sarcopenic and SMI of cachexia and control patients was similar, despite a significant difference in phase angle. Indeed, in patients with grossly altered body composition, a direct measure of muscularity with computed tomography or dual-energy X-ray is preferred, but not easily available in the clinical routine. The influence of hypogonadism, which is frequently observed in cachectic patients and contributes to accelerated bone remodeling in the elderly, should also be evaluated in follow-up studies [46]. Despite limitations, our data indicates profound changes in bone metabolism due to cachexia in cancer patients. Interestingly, ongoing research is focusing on the effect of bone targeted drugs to alleviate symptoms of cachexia, such as muscle wasting [47–49].

## Conclusions

In conclusion, our study provides evidence for a negative bone remodeling balance in cancer cachexia and supports further research not only to assess the risk of osteoporosis and fracture in this fragile patient population, but also to prevent bone loss and perhaps overcome cachexia-induced muscle wasting using bone modifying agents.

## Supplementary Information

**Additional file 1.**

## Data Availability

To preserve patient confidentiality the datasets generated for this study are not publicly available, but will be provided by the corresponding author on reasonable request.

## Abbreviations

25(OH)D: 25-hydroxy vitamin D
ALP: Alkaline phosphatase
BCM: Body cell mass
BIA: Bioimpedance analysis
BMI: Body mass index
BTMs: Bone turnover markers
Ca: Calcium
CHE: Cholinesterase
CRP: C-reactive protein
CTX: Carboxy terminal telopeptide of collagens
ECW: Extracellular water
eGFR: Estimated glomerular filtration rate
GGT: Gamma glutamyl transferase
ICW: Intracellular water
PINP: Procollagen type I N-terminal propeptide
PTH: Parathyroid hormone
PTHrP: Parathyroid hormone related protein
Ocn: Osteocalcin
SD: Standard deviation
SOPs: Standard operating procedures
TSH: Thyroid stimulating hormone
