# Supplementary material for: Effect of cachexia on bone turnover in cancer patients: a case-control study
Source: BMC Cancer. 2021 Jun 28;21:744. doi: 10.1186/s12885-021-08518-9 (PMC8240310; doi:10.1186/s12885-021-08518-9)
Supplement: Supplementary file 1 — Additional file 1. [file 12885_2021_8518_MOESM1_ESM.docx]

**Supplementary Table 1. Markers of bone turnover according to tumor type and disease stage, after exclusion of patients with bone metastases**

|  | **Tumor type** | |  | **Disease Stage** | | |  |
| --- | --- | --- | --- | --- | --- | --- | --- |
|  | **Lung Cancer** **(n=33)** | **Non-Lung Cancer (n=11)** | **p** | **I, II (n=14)** | **III (n=11)** | **IV** **(n=19)** | **p** |
| **CTX, ng/mL** | 0.37  (0.27-0.57) | 0.3  (0.28-0.3) | 0.3 | 0.3  (0.26-0.4) | 0.27  (0.2-0.4) | 0.4  (0.28-0.6) | 0.2 |
| **Ocn ng/ml** ^*^ | 15.7 (± 5.1) | 13.2 (± 5.4) | 0.2 | 16.9 (± 4.8) | 12.7 (± 5.1) | 15.1(± 5) | 0.1 |
| **CTX/Ocn ratio > 0.022, n (%)** | 15 (45.5) | 7 (63.6) | 0.3 | 5 (35.7) | 5 (45.5) | 12 (63.2) | 0.3 |
| **PINP, µg/L** | 28.3  (21-37) | 32.7  (23-39) | 0.4 | 29.4  (22-43) | 24.8  (21-30) | 32.7  (26-39) | 0.9 |
| **CTX/PINP ratio > 0.011, n (%)** | 17 (51.5) | 5 (45.5) | 0.7 | 6 (42.9) | 8 (45.5 | 11 (57.9) | 0.6 |

**Abbreviations:** CTX, carboxy terminal telopeptide of collagen type I; Ocn, osteocalcin; PINP, procollagen type I N-terminal propeptides.

**Notes:**

**-** all data are expressed as median and interquartile range (25-75th percentile), except for ^*^ Ocn (mean ± SD).

**Supplementary Table 2. BTM correlations with anthropometric, BIA and laboratory parameters after exclusion of patients with bone metastases (n=44).**

|  | **CTX** | | **Ocn** | | **PINP** |  |
| --- | --- | --- | --- | --- | --- | --- |
|  | **r** | ***p*** | ***r*** | ***p*** | ***r*** | ***p*** |
|  |  |  |  |  |  |  |
| **Anthropometric and BIA parameters** | | | | |  |  |
| **Age** | -0.008 | 0.9 | -0.2 | 0.17 | -0.04 | 0.8 |
| **BMI** | -0.27 | 0.07 | 0.17 | 0.27 | -0.2 | 0.2 |
| **Weight loss** | ***0.41*** | ***0.006*** | -0.24 | 0.11 | 0.04 | 0.8 |
| **Weight** | -0.28 | 0.06 | 0.15 | 0.3 | -0.17 | 0.2 |
| **Waist circumference** | ***-0.36*** | ***0.02*** | -0.02 | 0.9 | ***-0.34*** | ***0.02*** |
| **Fat mass** | -0.27 | 0.07 | 0.08 | 0.6 | -0.19 | 0.2 |
| **Total body water** | -0.2 | 0.1 | 0.06 | 0.6 | -0.08 | 0.6 |
| **ECW/ICW** | -0.07 | 0.6 | -0.13 | 0.4 | -0.06 | 0.7 |
| **BCM** | -0.15 | 0.3 | ***0.3*** | ***0.03*** | 0.03 | 0.8 |
| **Phase angle** | -0.22 | 0.1 | 0.23 | 0.1 | -0.003 | 0.9 |
| **SMI** | -0.05 | 0.8 | -0.05 | 0.7 | 0.04 | 0.8 |
|  |  |  |  |  |  |  |
| **Laboratory parameters** | |  |  |  |  |  |
| **eGFR** | 0.07 | 0.6 | 0.14 | 0.3 | 0.03 | 0.8 |
| **CRP** | ***0.38*** | ***0.009*** | -0.1 | 0.5 | 0.13 | 0.4 |
| **Albumin** | ***-0.42*** | ***0.004*** | 0.19 | 0.2 | -0.18 | 0.2 |
| **Cholinesterase** | ***-0.38*** | ***0.009*** | 0.3 | 0.08 | -0.23 | 0.1 |
| **ALP** | 0.2 | 0.15 | 0.06 | 0.7 | 0.15 | 0.3 |
| **GGT** | 0.4 | 0.05 | -0.1 | 0.4 | 0.07 | 0.6 |
| **Hemoglobin** | -0.29 | 0.05 | 0.1 | 0.5 | ***-0.34*** | ***0.02*** |
| **Lymphocytes** | -0.09 | 0.5 | 0.1 | 0.3 | -0.18 | 0.2 |
|  |  |  |  |  |  |  |
| **Bone metabolism parameters** | | | |  |  |  |
| **PTH** | -0.07 | 0.6 | 0.07 | 0.6 | -0.11 | 0.5 |
| **25(OH)D** | -0.06 | 0.6 | 0.17 | 0.3 | 0.1 | 0.5 |
| **TSH** | 0.25 | 0.09 | -0.12 | 0.4 | 0.09 | 0.6 |

**Abbreviations:** BTM, bone turnover markers; BIA, bioimpedance analysis; CTX, carboxy terminal telopeptide of collagen type I; Ocn, osteocalcin; PINP, procollagen type I N-terminal propeptides; BMI, body mass index; ECW, extracellular water; ICW, intracellular water; BCM, body cell mass; SMI, skeletal muscle index; eGFR, estimated glomerular filtration rate; CRP, C-reactive protein; ALP, alkaline phosphatase; GGT, gamma glutamyl transferase; 25(OH)D, 25-hydroxy vitamin D.
